# Entanglement-Mediated Dispersion of Lignin Nanoparticles in PVA Networks for Transparent and Tough Bio-Composites

**DOI:** 10.3390/polym18060691

**Published:** 2026-03-12

**Authors:** In Jun Lee, So Youn Kim

**Affiliations:** 1 Department of Chemical and Biological Engineering, Institute of Chemical Processes, Seoul National University, Seoul 08826, Republic of Korea; injun.lee@snu.ac.kr; 2 Hanwha Solutions Chemical Division, Research and Development Center, Daejeon 34128, Republic of Korea

**Keywords:** lignin nanoparticle, polymer nanocomposite, rheology, SAXS, dispersion stability, optical property

## Abstract

Lignin nanoparticles (LNPs) offer sustainable alternatives to petroleum-derived nanofillers, yet their industrial application remains limited by poor dispersion control and trade-offs between loading, optical clarity, and mechanical performance. Here, we present a molecular architecture-driven design framework that systematically decouples polymer network physics from nanoparticle dispersion in poly(vinyl alcohol)/LNP nanocomposites. Through eco-friendly self-precipitation, we synthesize uniform LNPs with size tunability, overcoming persistent reproducibility challenges. Systematic investigation across PVA molecular weights and LNP loadings reveals entanglement-controlled dispersion behavior. Combined rheological and small-angle X-ray scattering analyses demonstrate that macroscopic suspension rheology is governed exclusively by polymer chain overlap, remaining invariant across LNP loadings. Conversely, the nanoscale LNP microstructural organization—ranging from depletion-driven clustering in weakly entangled networks to network-confinement stabilization in densely entangled systems—fundamentally dictates the film’s optical clarity and mechanical toughness. This rheology-microstructure decoupling establishes critical processing windows for industrial formulations, where polymer entanglement ensures suspension processability while the LNP dispersion state enables optical–mechanical tunability. The entangled network’s structure-filtering effect provides robust protocols for fabricating sustainable, transparent bio-composites suitable for packaging, optics, and functional films. Our quantitative composition–structure–performance framework advances fundamental understanding of entanglement-mediated interfacial phenomena while delivering practical design rules for next-generation sustainable bio-composites.

## 1. Introduction

The effective utilization of lignin from lignocellulose waste streams represents a critical challenge in sustainable materials science. Annually, billions of tons of wood waste and agricultural residues are generated globally, with lignin—the second most abundant natural polymer after cellulose—typically being underutilized or combusted for energy recovery [1,2]. Lignin constitutes from approximately 20 to 35 wt% of lignocellulose biomass and possesses inherent aromatic structures, phenolic hydroxyl groups, and intrinsic stiffness that render it chemically and mechanically valuable [3,4,5,6]. However, the industrial implementation of lignin-based nanofillers remains hindered by several persistent challenges: (1) the structural complexity [7,8] and compositional variability of native lignin limit reproducible material synthesis, (2) poor control over nanoparticle size [2,9] and size distribution complicates both characterization and composite optimization, and (3) an inadequate understanding of lignin nanoparticle (LNP) dispersion mechanisms and interfacial interactions with polymer matrices has limited the rational design of composites with tailored multiscale structure and performance.

This study presents a systematic investigation of the synthesize of uniform lignin nanoparticles (LNPs) via an eco-friendly self-precipitation method [10,11,12,13] and their integration into polyvinyl alcohol (PVA) nanocomposites. PVA was selected as the model matrix polymer because: (i) it is biodegradable and non-toxic, making it attractive for sustainable [14,15] and biomedical applications [16,17]; (ii) its aqueous solubility enables straightforward solution casting; (iii) extensive hydrogen bonding in PVA chains provides high crystallinity and mechanical strength while maintaining optical transparency [18]; and (iv) PVA has been employed in prior lignin nanocomposite studies, enabling direct literature comparison [19,20,21].

A central objective of this work is to elucidate how polymer chain architecture—specifically, the molecular weight (MW) and resulting entanglement density of PVA—governs LNP dispersion state, aggregation behavior, and interfacial interactions in suspension and film states. Fundamentally, the dispersion of nanoparticles in polymer solutions is dictated by the polymer physics governing colloidal stability. As the polymer concentration exceeds the critical overlap concentration (c*), the system transitions into the semi-dilute and entangled regimes. In these regimes, nanoparticle organization is controlled by a delicate thermodynamic competition between macromolecular entanglements and inter-particle interactions. Short, weakly entangled chains often induce depletion-driven attraction, leading to local particle clustering, whereas long, densely entangled networks provide robust steric stabilization and kinetic confinement that suppress aggregation [22,23,24,25]. Recent literature has systematically highlighted how this entanglement density fundamentally governs nanoparticle organization in polymer media, and how such microstructural states manifest in distinct rheological signatures [26]. However, a critical knowledge gap remains regarding how these entanglement-mediated dispersion principles apply to complex, hydrogen-bonding bio-nanofillers like LNPs, and whether the macroscopic processability can be independently controlled from the nanoscale functional properties. Addressing this gap requires understanding the phenomenon of rheology–microstructure decoupling, where bulk viscosity is dominated exclusively by polymer chain overlap (c/c*), while the optical and mechanical performances are dictated by the localized nanoparticle dispersion state. By comparing two PVA grades of distinctly different molecular weights, we aim to map the relationship between polymer molecular weight, LNP aggregation phenomena, and emergent multiscale composite structure.

We found a decoupling between macroscopic process ability and microstructural evolution: bulk rheological properties in the dilute to semi-dilute regime are predicted to be governed primarily by polymer chain density and entanglement, as will be demonstrated through power-law analysis of viscosity scaling [27,28,29,30], such that moderate variations in LNP dispersion state may have limited impact on macroscopic flow resistance.

However, given that the synthesized nanoparticles are relatively small and, as will be shown later, sparsely distributed, LNPs may act primarily as nucleation sites or modifiers of polymer crystallization [31,32] rather than as dominant structural reinforcements. Furthermore, their arrangement and aggregation state are anticipated to have a pronounced influence on light scattering and optical responses such as haze and clarity [33,34]. Precise control of LNP dispersion, while not strictly required to maintain acceptable bulk mechanical performance, becomes critically important for applications where optical transparency or controlled scattering is essential.

This dual behavior motivates a comprehensive characterization strategy encompassing suspension-state rheology and small-angle X-ray scattering (SAXS), film-state structure analysis via SAXS and X-ray diffraction (XRD), and assessment of optical and mechanical performance. This integrated approach enables the correlation of nanoscale dispersion state with macroscopic composite behavior, yielding design principles for engineering polymer–lignin nanocomposites with optimized multiscale architecture.

In this work, we show the accomplishment of three primary objectives: (1) to synthesize uniform, small-sized LNPs (diameter < 50 nm) via eco-friendly self-precipitation and to characterize their core structure, hydrodynamic size, and synthetic tunability; (2) to establish a composition-dependent aggregation map across PVA concentrations (3 to 6 wt%) and LNP loadings (0 to 15 wt%), quantifying how polymer chain overlap concentration (c/c*) modulates the balance between steric repulsion and depletion-driven attraction; and (3) to assess the impact of LNP incorporation and dispersion state on nanoscale structure (via SAXS and XRD) and macroscopic properties (optical clarity and haze, mechanical tensile response) of PVA/LNP nanocomposites. By linking suspension-state molecular-level insights (chain mobility and entanglement) to film-state multiscale structure and final material performance, this study provides both fundamental understanding of polymer–nanoparticle interactions and practical design guidelines for developing high-performance, sustainable bio-based nanocomposites.

## 2. Materials and Methods

### 2.1. Materials

Polyvinyl alcohol (PVA) with weight-average molecular weights (M_w_, as specified by the manufacturer) of 31,000–50,000 g/mol (denoted as 40k) and 89,000–98,000 g/mol (denoted as 90k) was purchased from Sigma-Aldrich (St. Louis, MO, USA). Kraft lignin (commercial product Lineo™) was supplied by Stora Enso (distributed by Scranton Limited, Seoul, Republic of Korea). Acetone (purity ≥ 99.5%) was purchased from Daejung Chemicals and Metals (Siheung, Republic of Korea). Deionized (DI) water was used for all experiments.

### 2.2. Synthesis of Lignin Nanoparticles

**Figure 1:**
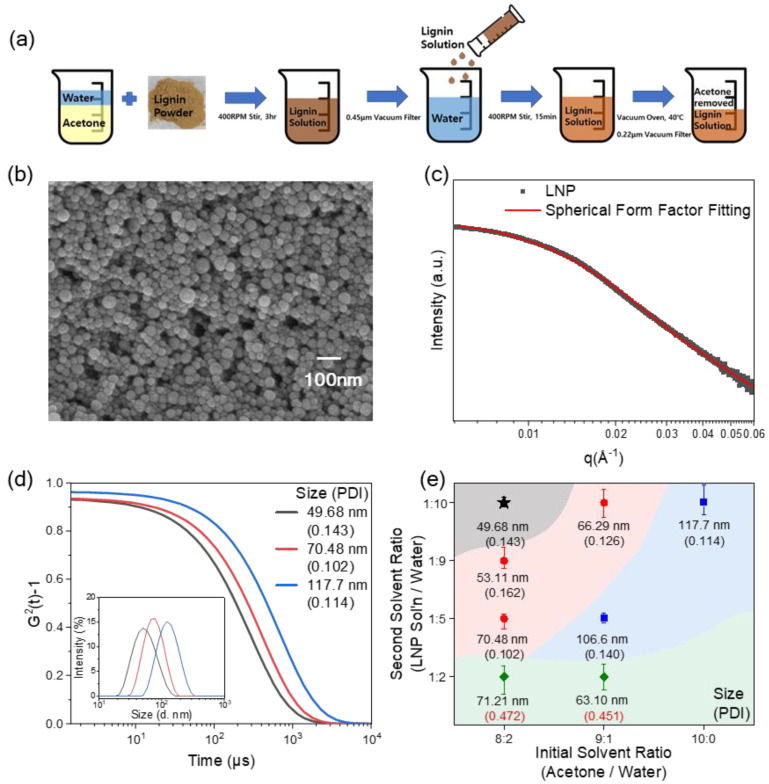
Synthesis and structural characterization of uniform LNPs. (**a**) Schematic illustration of the eco-friendly self-precipitation process. (**b**) SEM image of reference LNPs (35.2 ± 8.62 nm diameter). (**c**) SAXS spherical form-factor fit (33.44 nm diameter). (**d**) DLS correlation functions for varied conditions. (**e**) LNP diameter size (PDI) vs. solvent ratios (black star: reference).

Lignin nanoparticles (LNPs) were synthesized via a self-precipitation method using a solvent mixture of acetone and deionized water. Briefly, lignin (5 mg/mL) was dissolved in an acetone/water mixture (80:20 *v*/*v*) and stirred at 400 rpm for 3 h. The solution was vacuum-filtered using a bottle-top filter with a 0.45 μm pore size (polyethersulfone membrane) to remove undissolved impurities. A negligible amount of residue was observed on the filter membrane, confirming the near-complete dissolution of the lignin precursor. This aggregate-free precursor solution ensured homogeneous nucleation during the subsequent self-precipitation, which is retrospectively validated by the narrow size distribution and low polydispersity index (PDI) of the resulting LNPs observed in our DLS measurements. Subsequently, the filtered lignin solution was poured into deionized water (antisolvent) at a volume ratio of 1:10 (lignin solution to water) and stirred at 400 rpm for 12 min. The resulting suspension was dried in a vacuum oven at 40 °C for 12 h to remove acetone. The final aqueous LNP dispersion (concentrated to approx. 0.5 mg/mL) was filtered through a 0.22 μm syringe filter. For specific experiments requiring size control, the particle size was tuned by adjusting the initial solvent ratio and secondary mixing ratio. LNPs with diameters ranging from 40 to 120 nm were synthesized for subsequent experiments; however, LNPs with a diameter below 50 nm (Black-star marked in Figure 1e) were primarily used for PNC fabrication unless stated otherwise. This strict size threshold (<50 nm) was prioritized not only to ensure that the characteristic structural features fall optimally within the accessible q-range of SAXS measurements but also to minimize Rayleigh scattering, thereby preserving high optical transparency in the final nanocomposite films. The synthesized LNPs were imaged using scanning electron microscopy (SEM, Supra 55VP, Carl Zeiss, Oberkochen, Germany) at an accelerating voltage of 2.0 kV after coating the samples with platinum. Zeta potential measurements were conducted using a Zetasizer (ZS-90, Malvern Panalytical, Malvern, UK).

### 2.3. Preparation of Polymer Nanocomposite (PNC) Suspensions and Films

Polymer nanocomposites (PNCs) were fabricated via a solution blending and casting method using PVA with molecular weights of 40k and 90k combined with LNPs. First, a stock solution of PVA was prepared by dissolving PVA in deionized water at a concentration of 10 wt% at 90 °C for 2 h under stirring at 600 rpm. Two distinct series of suspensions were then formulated depending on the intended characterization:

(1) Constant PVA Concentration: While PVA concentration in the suspension was kept fixed (at specific concentrations, e.g., 3, 4, 5, and 6 wt%), LNPs were added at 0 to 15 wt% relative to the PVA mass. In this series, the total solid content increased slightly with LNP addition, but the polymer background concentration remained constant. The mixtures were stirred vigorously immediately after blending to ensure homogeneity. These stable PNC suspensions exhibited no phase separation or aggregation and were used directly for suspension-state characterizations, including rheology, DLS, and SAXS.
(2) Constant Total Solid Content: To ensure consistent film formation kinetics and uniform specimen dimensions for quantitative optical and mechanical analysis, the total solid concentration (PVA + LNP) was fixed at 5 wt%. The stock PVA solution was mixed with the LNP dispersion and additional deionized water, adjusting the PVA-to-LNP ratio to achieve LNP loadings of 0, 1, 5, 10, and 15 wt% relative to the total solids. This constant-solid approach allows for the systematic evaluation of bulk property modulation solely as a function of LNP volume fraction. The mixtures were stirred vigorously immediately after blending to ensure homogeneity, and no phase separation or aggregation was observed before and after film formation. The prepared 5 wt% suspensions were then cast into glass Petri dishes (diameter 7.8 cm) and dried in a vacuum oven at 40 °C for 24 h to ensure the complete evaporation of the aqueous medium and the elimination of any potential trace volatile solvents (e.g., residual acetone), thereby preventing solvent-induced plasticization. This process yielded uniform, freestanding films with a thickness of 100 ± 5 μm.

Sample compositions are primarily described in terms of weight percentage (wt%). However, for rheological modeling and scattering analysis where spatial occupancy is critical, these values were converted to volume fractions (ϕ_c_) using the measured densities of 1.19 g/cm^3^ for PVA and 1.35 g/cm^3^ for LNP.

### 2.4. Rheological Measurements

Rheological properties of PNC suspensions were measured using a rotational rheometer (MCR 302, Anton Paar, Graz, Austria) equipped with a concentric cylinder geometry (CC10). All samples were subjected to vortex mixing prior to measurement to ensure a well-dispersed state. The measurements were conducted at 20 °C, and shear viscosity was recorded over a shear rate range from 1 to 1000 s^−1^.

### 2.5. Small Angle X-Ray Scattering (SAXS)

Small angle X-ray scattering (SAXS) experiments were performed at the 4C SAXS II beamline of the Pohang Accelerator Laboratory (PAL, Pohang, Republic of Korea) to investigate the microstructure of LNPs within the PNCs. The accessible scattering vector range was 0.006 Å^−1^ < q < 0.1 Å^−1^, q = (4πsin θ)/λ, where θ represents half of the scattering angle and λ is the wavelength, 0.734 Å (SDD 5 m, 16.9 keV). Suspension samples were loaded into quartz capillary cells with a length of 80 mm, outer diameter of 1.5 mm, and wall thickness of 0.01 mm. For film samples, freestanding films were mounted directly on the sample holder. Background intensities, measured from empty capillaries for suspensions and air for films, were subtracted from the sample scattering intensities. The exposure time for each SAXS measurement was 30 s for suspensions and 10 s for films. The scattered intensity, I(q) can be written as, I(q) = ϕ_c_V_c_Δρ2P(q)S(q) + B, where V_c_ is the particle volume, Δρ is the difference in electron scattering length density between LNPs and the PVA matrix, P(q) is the form factor of LNPs, S(q) is the structure factor of LNPs, and B is the background. Due to the large electron density contrast (ρ_LNP_~1.79 to 1.97 × 10^–6^ Å^–2^ and ρ_PVA_~0.67 × 10^–6^ Å^–2^), the scattering intensity is dominated by LNPs after background subtraction. SAXS data were analyzed using Igor Pro 7.08 64-bit version software. For dilute suspensions, the scattering intensity was fitted to a spherical form-factor model incorporating polydispersity.

### 2.6. Fourier Transform Infrared Spectroscopy (FT-IR)

Fourier transform infrared (FT-IR) spectra were acquired using a spectrometer (Tensor 27, Bruker, Billerica, MA, USA) located at the National Center for Interuniversity Research Facilities (NCIRF) at Seoul National University. Measurements were taken over a wavenumber range from 4000 to 400 cm^−1^ with a spectral resolution of 1 cm^−1^. Each spectrum represents the average of 64 scans to ensure data reliability.

### 2.7. X-Ray Diffraction (XRD)

Crystalline structures of the PNC films were analyzed using an X-ray diffractometer (SmartLab, Rigaku, Tokyo, Japan) equipped with a Cu Kα source. Scans were performed at room temperature over a 2θ range of 10° to 60° with a scan rate of 0.6°/min. The degree of crystallinity (X_c_) was calculated by deconvolution of the crystalline and amorphous peaks using Gaussian functions with baseline correction from the diffraction profiles, according to X_c_ = A_c_/(A_c_ + A_a_), where A_c_ and A_a_ are the integrated areas of crystalline and amorphous regions, respectively.

### 2.8. Optical Properties

The optical properties, including total transmittance, haze, and clarity, of the PNC films were measured using a haze meter (Haze-gard plus, BYK-Gardner, Geretsried, Germany) in accordance with ASTM D1003 standards [35]. Measurements were taken at five different locations on each film, and the average values are reported. To visualize the crystalline morphology and mesoscale texture, polarized optical microscopy (POM) images were acquired using an optical microscope (BX53, OLYMPUS, Tokyo, Japan). The films were observed under cross-polarizers using a 10× objective lens (total magnification of 100×) to detect birefringence arising from ordered crystalline domains.

### 2.9. Thermal Analysis

Thermogravimetric analysis (TGA) was performed using a thermogravimetric analyzer (TGA5500, TA Instruments, New Castle, DE, USA) to evaluate thermal stability. Samples were heated from room temperature to 750 °C at a heating rate of 10 °C/min under a nitrogen atmosphere.

### 2.10. Dynamic Mechanical Analysis (DMA)

Mechanical properties were evaluated using a Dynamic Mechanical Analyzer (DMA Q800, TA Instruments) in tension film mode. Rectangular specimens with dimensions of 20 mm (length) × 7 mm (width) × 0.1 mm (thickness) were prepared. Samples were mounted on the tension clamps, and a pre-strain of 0.1% was applied to ensure sample tautness and proper alignment prior to testing at room temperature. Subsequently, a strain ramp was applied at a rate of 50% per minute up to failure or 300% strain. The secant modulus was calculated from the stress–strain slope at 4% strain to account for the non-linear elastic behavior of PVA. Toughness was determined by integrating the area under the stress–strain curve up to the 300% strain limit for all samples to ensure a consistent comparison of energy-dissipation capacity. Four replicates were tested for each composition to ensure reproducibility.

## 3. Results and Discussions

### 3.1. Preparation of Lignin Nanoparticles (LNPs)

We first present a synthesis of uniform LNPs. LNPs were synthesized via an eco-friendly self-precipitation method using a binary solvent system (water/acetone). This approach avoids toxic organic solvents and enables quantitative control of particle size distributions. In contrast to conventional lignin particle preparation routes that often produce micron-sized, irregular particles—undesirable for high-performance applications—our method successfully produced uniform, spherical LNPs. For the subsequent composite studies, we primarily utilized LNPs with diameters below 50 nm (as shown in Figure 1), which maximize specific surface area for polymer interaction while allowing precise SAXS structural characterization and minimizing optical scattering in the final composites.

Figure 1a shows the schematic illustration of the LNP preparation process: lignin powder is dissolved in an acetone/water mixture under stirring, the resulting lignin solution is purified by vacuum filtration and then poured into deionized water to induce self-precipitation; after removal of acetone and secondary filtration, a stable aqueous LNP dispersion is obtained (see Section 2 for details) By adjusting the initial solvent ratio of acetone to water and subsequently controlling the secondary dilution ratio (lignin solution/water), a self-precipitation method was employed to rapidly reach a critical concentration that governs nanoparticle growth. This approach ultimately yielded uniform, small-sized LNPs.

The characterization of the nanoparticles was performed using scanning electron microscopy (SEM), dynamic light scattering (DLS), and SAXS form-factor analysis. Figure 1b shows the SEM images of LNPs prepared under the standard solvent condition: an initial acetone/water volume ratio of 8:2, and a secondary lignin solution/water volume ratio of 1:10 (denoted as the reference condition and marked by the black star in Figure 1e), confirming the successful production of nearly uniform and spherical particles of LNPs. The particle size was determined to be 35.2 ± 8.6 nm based on SEM analysis of 50 individual LNPs.

SAXS provides a more reliable estimate of the core particle size than DLS because it is insensitive to the solvated layer. The intensity profile shown in Figure 1c is fitted with the form factor of spherical particles considering polydispersity. P(q, R) = [3{sin(qR) − qRcos(qR)}/(qR)^3^]^2^, where R is the particle radius, averaged over a narrow Gaussian radius distribution. The fitted SAXS form-factor analysis yielded a particle diameter of 33.44 nm with a standard deviation of 0.35.

We emphasize that the size of LNPs can be systematically varied by controlling the initial and second solvent ratios. Decreasing the water content in the second solvent ratio at a fixed initial solvent composition leads to a gradual increase in particle diameter and, beyond a certain threshold, to broader size distributions accompanied by higher PDI.

In Figure 1e, we present the variation of the size and PDI of LNPs. At a fixed secondary solvent ratio, increasing the acetone content in the initial solvent ratio also promotes particle growth, which determines the final size of the LNP. These trends are highly reproducible, demonstrating that the solvent composition provides a robust handle to tailor the LNP size by DLS from about 50 nm particles to larger, depending on the intended application.

The sizes of LNPs were measured by DLS. Figure 1d shows the correlation functions of LNPs synthesized from different conditions; for example, the sample (black) exhibited a mean particle diameter of 49.68 nm (inset) with PDI of 0.143. The diameter size of LNP can be increased up to 117 nm with moderate variation of PDI. The difference between DLS and SAXS measurements is attributed to the hydrodynamic diameter measured by DLS, which accounts for the solvent layer and possible aggregates, resulting in larger apparent sizes. Importantly, this pronounced size discrepancy implies the presence of a thick, strongly bound hydration shell around the LNPs due to hydrogen bonding with water. This hydration layer acts as a robust physical barrier that effectively prevents the solid cores from making direct contact, thereby imparting excellent long-term colloidal and dispersion stability to the LNPs in aqueous media. The synthesized LNP is highly dispersible in water due to its electrostatic repulsions. To understand the underlying stabilization mechanisms, the electrostatic properties of LNPs were characterized. Zeta potential measurements of lignin solutions in Figure S1 without PVA show values of −33 to −19 mV, indicating that LNPs carry a significant negative surface charge [11] and are electrostatically stabilized in aqueous media. However, in the presence of non-ionic polymers such as PVA, this electrostatic stabilization may be compromised due to charge screening or adsorption effects, necessitating a systematic investigation into the dispersion stability within the polymer matrix.

### 3.2. LNP Aggregation Map in PVA Solutions

**Figure 2:**
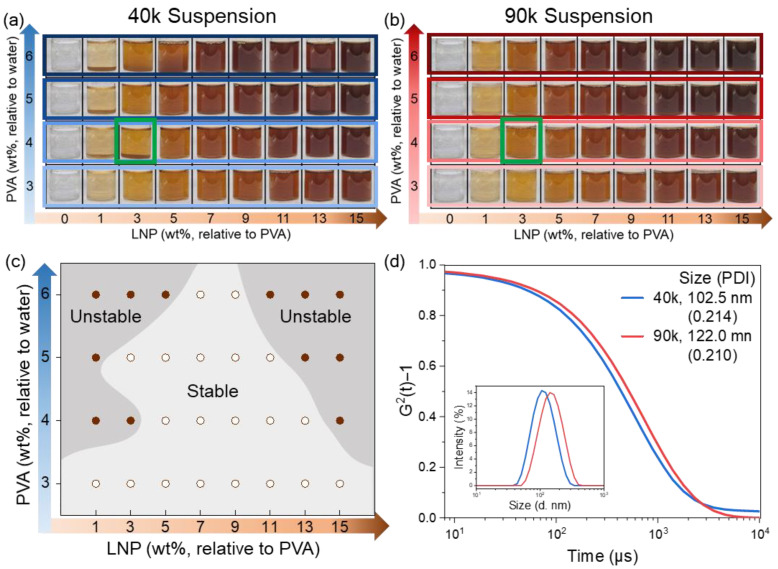
Dispersion behavior and stability of LNPs. (**a**,**b**) Optical images of (**a**) 40k and (**b**) 90k after 24 h. (**c**) Aggregation phase diagram summarizing stability boundaries for 40k. (**d**) DLS correlation functions at 5 wt% PVA system.

We next investigate how LNP dispersion stability depends on PVA molecular weight and formulation. PVA/LNP suspensions were prepared by varying PVA concentration (3, 4, 5, and 6 wt% in water) using two PVA grades (MW = 40k and 90k). For each PVA concentration, the LNP loading was systematically increased from 0 to 15 wt% relative to PVA. Optical images of the suspensions recorded 24 h after mixing are shown in Figure 2a (40k) and Figure 2b (90k). In Figure 2a,b, “LNP wt%” denotes wt% relative to PVA; for example, the sample in the green box contains 4 wt% PVA with 3 wt% LNP (relative to PVA) corresponds to an absolute LNP concentration of 0.12 wt% in water.

As expected, sample color darkens with increasing LNP content due to lignin’s intrinsic absorption. Most suspensions remain macroscopically stable, but distinct molecular-weight-dependent behavior emerges. For the 90k PVA system, all compositions remain visually homogeneous with no apparent sedimentation or phase separation across the explored range. In contrast, the 40k system is stable at low polymer concentration (3 wt%) but exhibits aggregation at both low and high LNP loadings when PVA concentration increases (Figure 2a), indicating a non-monotonic stability window.

This non-monotonic aggregation in 40k can be rationalized by the interplay of (i) depletion-driven attraction, (ii) steric stabilization via polymer adsorption, and (iii) polymer-to-particle ratio. At 3 wt% PVA, polymer concentration is insufficient to generate strong depletion attraction or substantial screening; thus, the native electrostatically stabilized LNP dispersion is largely preserved. As PVA increases (4–6 wt%), aggregation appears at low LNP loading (high polymer-to-particle ratio), where excess free polymer can generate a strong depletion attraction that drives compact aggregation. At intermediate LNP loadings, adsorption-mediated steric repulsion and reduced depletion driving force can balance each other, producing the most stable dispersion window (no visible aggregation). At high LNP loadings, the system tends to form looser, reversible clusters that redisperse upon gentle agitation, consistent with weaker effective attractions at a reduced polymer-to-particle ratio.

In contrast, the 90k system is far less sensitive to composition. The longer chains form a more entangled solution structure and provide thicker steric layers, which together (i) reduce collision frequency (kinetic stabilization) and (ii) increase the steric barrier (thermodynamic stabilization), suppressing macroscopic aggregation and sedimentation over the entire tested range.

Zeta potential measurements further support the finding that polymer addition weakens electrostatic stabilization and shifts the dominant physics toward polymer-mediated interactions. Upon adding PVA, the magnitude of the negative zeta potential decreases substantially (Figure S1), indicating charge screening and/or an altered interfacial charge environment. For example, for 90k PVA, ζ decreases from ~−33 mV (bare LNPs) to approximately −11.0, −4.9, and −3.0 mV at 1.5, 3.0, and 4.5 wt% PVA, respectively, while 40k reduces ζ even more strongly (to ~−2.9 to −1.3 mV at comparable conditions). These reductions imply that electrostatic repulsion is significantly weakened in the presence of PVA, thereby amplifying the role of depletion and steric effects in controlling aggregation.

To quantify the dispersed state, DLS measurements were performed for representative suspensions at 5 wt% PVA (Figure 2d). In both 40k and 90k systems, the apparent hydrodynamic size increases relative to bare LNPs, consistent with polymer adsorption and/or weak clustering. The 40k system shows an average aggregate size of ~100 nm with a narrow distribution, whereas the 90k system shows a slightly larger apparent size (~120 nm), consistent with a thicker polymer adsorption layer for higher MW PVA rather than stronger aggregation.

Long-term stability tests further support the molecular-weight-dependent stabilization mechanism. Over 72 h (Figure S2), 90k suspensions remain stable with no visible sedimentation, while 40k suspensions progressively exhibit aggregation and settling. Importantly, lignin dispersions without polymer remain stable even at high concentrations (Figure S1), confirming that aggregation observed in PVA/LNP systems originates from polymer-mediated interactions under screened electrostatic conditions. Overall, these results establish that polymer chain length is a key determinant of LNP kinetic stability in PVA solutions.

### 3.3. Rheological and SAXS Analysis

**Figure 3:**
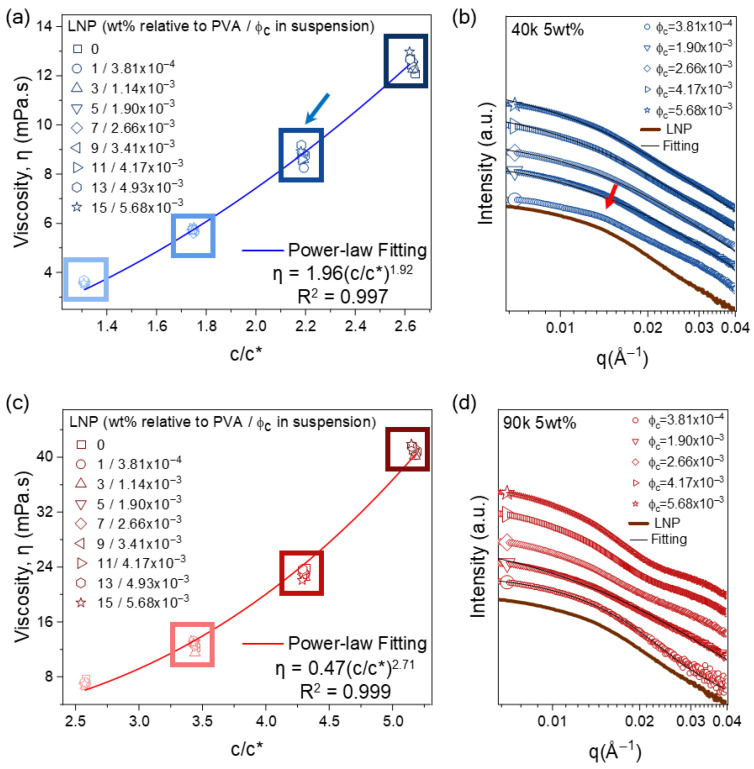
Shear viscosity and SAXS analysis decoupling polymer vs. LNP effects. (**a**,**c**) Shear viscosity vs. c/c* for (**a**) 40k and (**c**) 90k PVA system. (**b**,**d**) SAXS intensity at 5wt% PVA. Solid lines indicate spherical form-factor fits. (**b**) In the 40k system: At low ϕ_c_ peak (red arrow, clustering); form-factor fits give diameter (std. dev.); ▽ 27.2 nm (0.47), ◇ 29.0 nm (0.44), ▷ 32.6 nm (0.46) and ☆ 34.2 nm (0.45). (**d**) In the 90k system: ○ 33.2 nm (0.28), ▽ 29.2 nm (0.56). The fitting deviates at high ϕ_c_.

Next, we measured the shear viscosity of the PVA/LNP suspensions across the compositional range shown in Figure 2 (3 to 6 wt% PVA, 0 to 15 wt% LNP relative to PVA). All samples exhibited nearly Newtonian behavior within the measured shear rate range (1 to 1000 s^−1^, Figure S3); therefore, the viscosity at 100 s^−1^ is plotted against the normalized concentrations (c/c*) in Figure 3a,c. To calculate the theoretical critical overlap concentration (c* = M/[N_A_(4πR_g_^3^/3)]), the radius of gyration (R_g_) was theoretically estimated using polymer scaling principles. Specifically, the unperturbed dimension (R_g,0_) was initially derived based on the freely jointed chain model utilizing the median molecular weight of each PVA grade (vinyl alcohol monomer mass = 44.05 g/mol). To account for the excluded volume effect in a good solvent (water), the swollen R_g_ was calculated using the scaling relation R_g_ = R_g,0_ N^ν−0.5^, where N is the degree of polymerization and ν = 0.588 is the Flory exponent. Based on this estimation, c* was calculated as 22.91 g/L for 40k PVA and 11.65 g/L for 90k PVA. In Figure 3a,c, at the same c/c*, multiple points are plotted with increasing LNP concentrations (see the legend). For example, the viscosity of the samples in the blue boxes in Figure 2a is plotted in the same blue boxes in Figure 3a.

A key observation is that viscosity increases strongly with c/c* but remains essentially insensitive to LNP loading at fixed c/c* (within each “box” grouping). This is consistent with the extremely low LNP volume fraction (ϕ_c_ < 0.01): the classical hydrodynamic viscosity increment for rigid spherical particles is negligible at such low ϕ (a maximum relative viscosity increase of ~1.4% verified via the Huggins equation; see Note S1), and the dominant contribution arises from polymer overlap/entanglement. Moreover, the small and uniform LNPs can reside within the polymer mesh without forming a percolated filler network at these loadings; thus, bulk flow resistance is governed primarily by polymer chain physics.

The molecular-weight dependence is evident when comparing regimes. In 90k suspensions, the explored c/c* range (2.6–5.1) corresponds to a semi-dilute, densely entangled regime. The overlapped long chains form a robust viscoelastic matrix and provide thick steric barriers, maintaining high dispersion stability. In contrast, the 40k system operates closer to the entanglement threshold (c/c*~1.3–2.6), where steric stabilization is weaker and the reduced zeta potential further suppresses electrostatic stabilization. Consequently, 40k is more prone to depletion-driven clustering as polymer density increases, consistent with the polymer–colloid literature [36,37,38].

Even at the similar c/c* values, the markedly different aggregation behaviors between 40k and 90k systems demonstrate the role of chain length. The more extended steric barrier and thicker depletion layer formed by 90k chains creates a more favorable environment for maintaining LNP dispersion. Despite these different dispersion states, the changes in macroscopic rheological properties are subtle. Specifically, at a fixed polymer concentration (e.g., 5 wt% PVA, see the box with the blue arrow), the shear viscosity remains essentially constant regardless of LNP content (0 to 15 wt%, Figure 3a,c). Thus, the flow resistance is governed exclusively by polymer chain overlap and entanglement, independent of nanoscale LNP aggregation state. The viscosity differences observed between 40k and 90k systems arise from differences in c/c* and chain length, not from variations in the individual stability of LNP or its aggregate size.

The polymer-dominated rheological behavior is further quantified by power-law scaling, η~(c/c*)*^n^* where the exponent n reflects chain entanglement state and network density. According to classical polymer scaling theory for a good solvent [30,39], the viscosity scales with an exponent of 1.3 in the semi-dilute unentangled regime and 3.9 in the fully entangled regime. Our observed exponents fall within this intermediate crossover region. Specifically, the 40k system exhibits *n* = 1.92, indicating that the chains have crossed the entanglement threshold and entered a weakly entangled state. In contrast, the 90k system yields a higher exponent of *n* = 2.71, demonstrating that the longer chains have surpassed this threshold and are situated deeper within the entangled regime. Although the broad molecular-weight distribution of the commercial PVA precludes the exact calculation of absolute theoretical entanglement thresholds, these distinct scaling exponents internally validate the transition from a weakly to a densely entangled network, supporting our phenomenological interpretation. Importantly, these exponents—and the viscosity trends—are largely unaffected by LNP dispersion state at the explored ϕ_c_ range (up to 7 × 10^−3^), reinforcing that rheology is controlled by polymer overlap and entanglement rather than nanoparticle organization.

We performed SAXS experiments for PVA/LNP suspensions. The SAXS intensity profiles of LNP extracted from PVA/LNP suspensions reveal structural signatures depending on PVA molecular weight. In the 40k PVA system (Figure 3b), the scattering profile at the lowest ϕ_c_ exhibits a peak (arrow) corresponding to a characteristic correlation length of about 43 nm. This signature of local clustering, consistent with the optical images in Figure 2a, arises from the thin steric/depletion layers of short 40k PVA chains. At higher ϕ_c_, the I(q) curve resembles the spherical form factor of the bare LNP (introduced in Figure 1c), noting that the standard deviation becomes greater, (0.35 for bare LNP, 0.45 to 0.47 for LNP in polymer suspension), implying local aggregation and wider size distribution.

Even if the 40k PVA drives depletion attraction, inducing local particle clustering, the macroscopic rheology remains unaffected. This is fundamentally due to the very low LNP volume fraction (ϕ_c_ < 0.01). At such low filler loadings, these localized LNP clusters do not evolve into a robust, long-range percolated network; thus, the bulk viscosity is predominantly governed by polymer chain overlap and entanglement rather than filler–filler interactions. As a result, viscosity of the 40k system is governed primarily by increased polymer chain overlap (c/c*) concentration and modest particle–polymer bridging rather than particle-network reinforcement, as reflected by the lower power-law exponent (*n* = 1.92).

In the 90k system (Figure 3d), I(q) exhibits multiple curvatures over a broad q range, slightly deviating from the spherical form-factor scattering of the bare LNP. At low ϕ_c_, the scattering profiles similarly resemble the LNP form factor, suggesting that good dispersion. However, the deviation from the spherical form factor at high ϕ_c_, suggesting that inter-particle correlations (structure factor contributions) become significant under these conditions. The detailed form-factor fitting supports this view: low-ϕ_c_ suspensions described reasonable size distributions (average size of 30 to 32 nm in diameter with the standard deviation of 0.5), whereas high-ϕ_c_ suspensions could not be adequately described by the spherical form factor, yielding physically unrealistic parameters. Though the cumulative effect of steric hindrance and polymer-mediated inter-particle interactions persist in the 90k system, the LNP incorporation does not change the trend of viscosity increment of the 90k suspensions; the viscosity remains the same at the same c/c* despite the increasing LNP content.

Increased PVA molecular weight thus governs not only the viscosity scaling behavior but also the spatial arrangement and structural stability of LNPs. Importantly, despite the distinct microstructural rearrangement of LNPs (dispersion vs. aggregation), the bulk flow behavior is predominantly governed by the polymer physics (c/c*). This decoupling between microstructure and rheology, allowing for the tuning of optical/mechanical properties without compromising suspension processability of the PNCs.

### 3.4. Effect of PVA/LNP Ratio on Structure and Rheology in Suspensions

**Figure 4:**
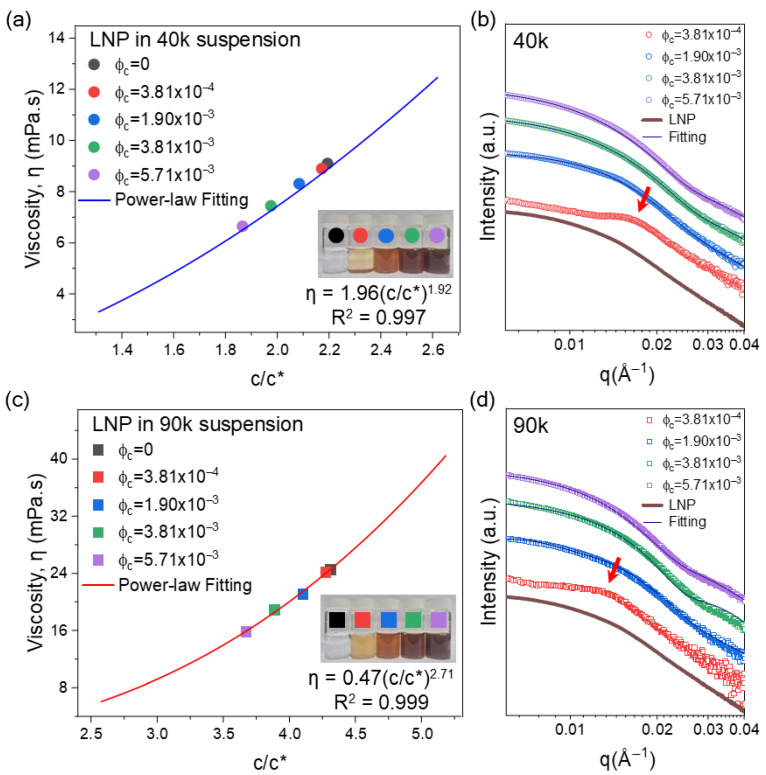
Effect of PVA/LNP ratio on rheology and nanoscale structure under constant-solid conditions. (**a**,**c**) Shear viscosity vs. c/c*. Insets show photographs of corresponding suspensions. (**b**,**d**) SAXS profiles showing structural changes. Red arrows indicate the characteristic correlation peak.

While the previous section (discussed with Figure 2 and Figure 3) examined the viscosity and structure by simply adding LNPs to fixed PVA contents (thereby increasing the total solid content from 3.03 to 6.9 wt%), practical industrial formulations often require optimizing the polymer–filler ratio at a fixed total loading. To address this, we isolated the effect of the PVA-to-LNP ratio by fixing the total solid content at 5 wt%. In this ‘constant-solid’ series, increasing the LNP loading inherently leads to a reduction in the PVA concentration, which consequently lowers the effective polymer chain density (c/c*). Figure 4a,c display the viscosity profiles of the 40k and 90k PVA systems, respectively, plotted against the reduced PVA concentration (c/c*) as the LNP fraction increases. As shown with the raw data in Figure S4a, all suspensions remain as nearly Newtonian liquids; therefore, the viscosity at 100 s^−1^ is plotted against the normalized concentrations.

For both molecular weights, decreasing PVA concentration (c/c*) leads to a reduction in viscosity that follows the power-law relationship η~(c/c*)*^n^* (*n* = 1.92 for 40k, 2.71 for 90k) established in Figure 3. These results confirm that, despite binding to polymer chains, LNPs do not impede polymer network formation and that viscosity is predominantly governed by polymer chain density and entanglement.

The 40k systems exhibited visible particle sedimentation at low LNP concentration conditions (red, ϕ_c_ = 3.8 × 10^−4^), as shown in the inset of Figure 4a, consistent with prior results showing that high PVA/LNP ratios promote short-range depletion-driven attraction of LNPs. In contrast, no visible particle aggregation or macroscopic precipitation was observed in the 90k systems, even at high LNP contents. Consistent with previous observations, the entangled network of high-molecular-weight PVA restricts inter-particle mobility and suppresses the depletion-driven aggregation, thereby providing sufficient stabilization.

SAXS measurements revealed that LNP organization depends sensitively on both PVA molecular weight and the PVA/LNP ratio. Figure 4b,d show the scattered intensity of the samples in Figure 4a,c, respectively. The detailed shape of the scattering curves could be described by a spherical form-factor model, highlighting different dispersion structures in the 40k and 90k systems, and indicating different mechanisms of particle organization.

In 40k systems, the lowest LNP content (red, ϕ_c_ = 3.8 × 10^−4^) shows a pronounced shoulder peak at q~0.18 Å^−1^ corresponding to a characteristic correlation length, about 35 nm. This dimension reflects the internal scale of local clusters driven by depletion attractions, which is also consistent with the particle sedimentation (inset of Figure 4a). At the intermediate LNP fraction (blue, ϕ_c_ = 1.9 × 10^−3^), the SAXS profile most closely matches the bare-LNP form factor, and the fitted diameter (32.4 nm) is very similar to those of neat LNPs, identifying this composition as a structurally well-dispersed, quasi-single-particle state.

Across the full LNP loading range in 40k systems, spherical form-factor fits remain acceptable, with effective diameters in the range 27 to 32 nm and only modest broadening of the size distribution (std. dev. 0.26 to 0.37) (see Figure S4b). This behavior indicates that although local clusters form at low LNP content, the short-chain 40k network does not develop strong, long-range particle connectivity, consistent with the lower rheological exponent *n* = 1.92 and the modest sensitivity of bulk viscosity to LNP loading.

In the 90k system, similar qualitative features appear but at systematically larger length scales. At low concentration (red, ϕ_c_ = 3.8 × 10^−4^), a shoulder peak near q~0.13 Å^−1^ appeared corresponding to a characteristic correlation length of ~49 nm. This expanded correlation length compared to the bare particle size suggests local clustering of LNPs, reflecting steric layers by the adsorbed long PVA chains with an entangled network, which maintains LNPs further apart and suppresses strong depletion-driven aggregation. As the LNP content increases to the intermediate composition (blue, ϕ_c_ = 1.9 × 10^−3^), the SAXS profiles approach the LNP form factor, and form-factor fitting yields an effective diameter comparable to that of pure LNP, signifying a more uniformly dispersed state dominated by excluded-volume interactions and steric repulsion.

In green (ϕ_c_ = 3.81 × 10^−3^) and purple (ϕ_c_ = 5.71 × 10^−3^) compositions, a notable difference emerges compared with the previous fixed-PVA experiments. When the PVA content was held at 5 wt% in Figure 3d, and only LNP was increased, high-ϕ_c_ suspensions could not be described by a simple spherical form factor, indicating that strongly entangled polymer networks drove dominant structure-factor contributions. In contrast, under the fixed-solids conditions, the same LNP volume fractions yield reasonable form-factor fits (Figure S4c), implying that the reduced PVA content weakens the network contribution, and thus that LNP structures behave more like a set of enlarged effective scattering objects.

At the highest LNP concentration (purple, ϕ_c_ = 5.71 × 10^−3^), the fitted effective diameter increases to 39.6 nm, indicating a shift of the scattering toward larger apparent length scales and the emergence of larger LNPs, presumably due to the adsorption and steric stabilization of long PVA chains. In contrast, the 40k system at the same LNP fraction remains describable by a form factor with only moderate broadening of bare LNPs.

Overall, these SAXS results reinforce the rheological interpretation: in 40k, a partial aggregation is found via depletion-driven local clustering in a weakly entangled network, confirmed with a lower power-law exponent, whereas in 90k, the LNPs are mainly dispersed with steric stabilization in the densely entangled matrix, which limits the mobility of LNPs. This is consistent with the higher exponent *n* = 2.71. Thus, increasing PVA molecular weight shifts the dominant inter-particle interaction from attraction-dominated clustering to entangled matrix-controlled dispersion, while the PVA/LNP ratio serves as a secondary control parameter that adjusts the balance between depletion-driven aggregation and steric stabilization; thus, molecular weight and LNP concentration dictate both nanoscale structure and macroscopic flow in LNP-based PNCs.

### 3.5. PNC Film Characterization

**Figure 5:**
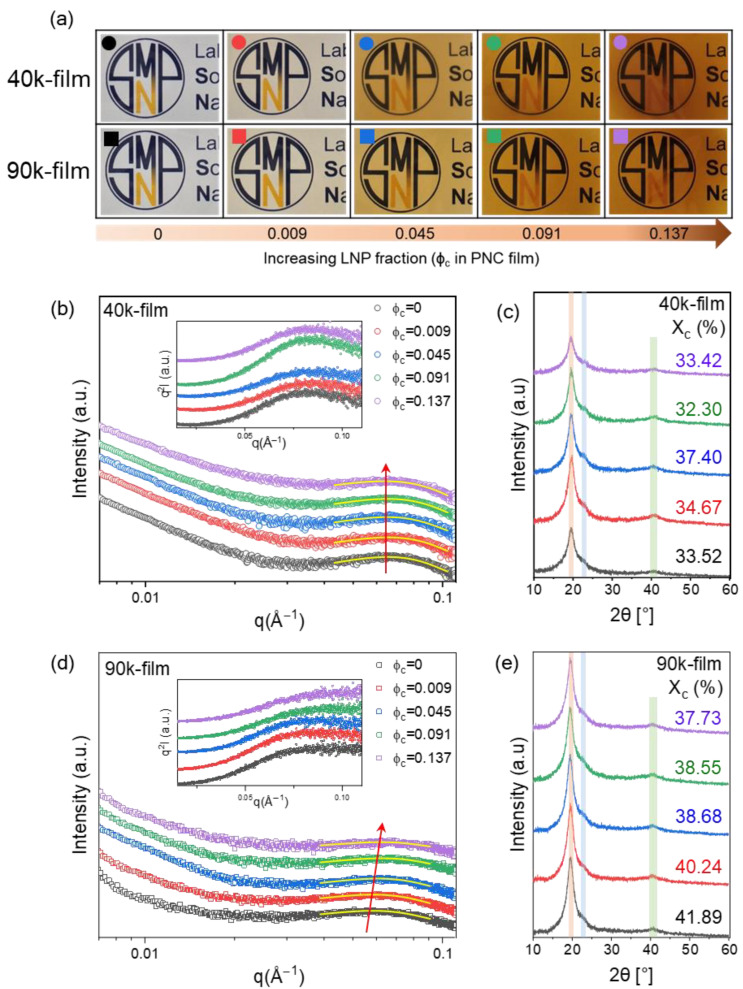
Macroscopic appearance and crystalline structure of PNC films. (**a**) Photographs of 40k (circle) and 90k (square) PNC films. (**b**,**d**) SAXS profiles indicate the domain spacing; insets display Lorentz-corrected profiles, q^2^I(q) to clearly visualize the primary scattering peak. The yellow lines represent Gaussian fitting used to determine the peak positions, and the red arrows indicate the shift of the scattering peaks with increasing LNP content. (**c**,**e**) XRD patterns and calculated crystallinity (X_c_).

Next, we investigated the effect of LNP dispersion on PNC films. The PNC films were prepared by casting PVA/LNP suspensions at the same PVA/LNP ratios used in Figure 4 into circular glass Petri dishes (diameter 78 mm) and drying to obtain free-standing films with a thickness of approximately 0.1 mm. The produced films are shown in Figure 5a. As the LNP fraction increased, the films gradually developed a more intense brown coloration, consistent with the intrinsic optical absorption of lignin, while remaining macroscopically uniform and free of visible phase separation. The colored symbol of each image in Figure 5 is matched with the suspensions investigated in Figure 4.

FT-IR spectra of the PNC films (Figure S5) were recorded in the O–H stretching region in the 3200–3400 cm^−1^ range and lignin aromatic and carbonyl vibrations in the 1650–1500 cm^−1^ range. The pure PVA films exhibit a broad O–H stretching band centered near 3300 cm^−1^, in agreement with typical PVA-based films reported in the literature [21,40,41], and no systematic peak shifts (≥10 cm^−1^) were observed in this region upon LNP incorporation for either 40k or 90k PVA system. In the 1650–1500 cm^−1^ range, the characteristic aromatic and carbonyl bands of lignin appeared and increased in intensity with LNP loading, confirming successful incorporation of LNPs into the PVA matrix without the emergence of new unexpected bands. These observations indicate that homogeneous PNC films were successfully fabricated across all compositions.

### 3.6. PNC Film Structure Analysis

Then, SAXS was used to evaluate the microstructure of the PVA network and its crystallization in PNC films [17]. Figure 5b,d show the scattered intensity of the PNC films with increasing LNP content for 40k and 90k PVA, respectively. To isolate the contribution of the polymer matrix, the neat 40k PVA film (black) was investigated as a baseline, exhibiting a characteristic peak at q = 0.0653 Å^−1^, which corresponds to a d-spacing of ~9.6 nm. In accordance with established scattering models for semi-crystalline PVA [41,42], this peak is attributed to the characteristic correlation length of the semi-crystalline morphology, representing features such as the lamellar long period or density fluctuations between crystalline and amorphous domains. Upon LNP addition, the peak position varies only slightly between 0.0635 and 0.0655 Å^−1^, indicating that the characteristic crystalline domain spacing is essentially preserved. This comparative observation, where the peak remains spatially stable regardless of LNP loading, suggests that the signal originates primarily from the intrinsic polymer matrix architecture rather than filler-induced ordering. The red arrows in Figure 5b visualize this shift toward higher q values, while the yellow lines represent the Gaussian fitting used to precisely determine these peak positions.

This insensitivity of the peak position to LNP content suggests that the loosely entangled 40k PVA network is not substantially reorganized by LNPs; the LNPs mainly form local clusters and only partially disturb the semi-crystalline morphology, behaving as fillers dispersed within a pre-existing network, consistent with the suspension-state SAXS and rheology results where 40k systems showed attraction-driven local clustering but limited network reinforcement.

Conversely, pure 90k PVA films displayed a characteristic peak at q = 0.0583 Å^−1^, corresponding to a larger d-spacing of approximately 10.8 nm. With increasing LNP fraction, this peak systematically shifts toward higher q values (up to 0.0627 Å^−1^), reducing d-spacing to approximately 10 nm. This indicates that the average spacing between semi-crystalline PVA domains and their intervening amorphous regions becomes slightly more compact. This gradual reduction in d-spacing shows that the crystallization of entangled PVA in the 90k film is influenced by LNP dispersions: polymer chains may rearrange and pack more tightly around LNPs under hydrogen bonding, leading to a more tightly structured crystalline–amorphous architecture. To further evaluate the crystalline structure of PNC films, XRD analysis [43,44] was conducted, and all films showed a characteristic PVA diffraction peak near 2θ~20°.

In the 40k system, the crystallinity remained relatively constant with increasing LNP content, varying within a narrow range (33 to 37%) and showing a modest maximum at the intermediate lignin loading (ϕ_c_ = 0.045, X_c_ = 37.40%). Therefore, since this variation is considered to be within the typical margin of error for XRD peak deconvolution, these results represent a state of structural stability rather than a definitive reinforcing trend. At this content, the PVA chains were largely concentrated (c/c*~2); thus, LNP may provide extra nucleation sites, slightly enhancing the overall crystallinity. In contrast, the 90k system showed substantially higher baseline crystallinity (X_c_ = 41.89% at pure PVA). Although a slight decrease to 37.73% was recorded at the highest loading, this shift does not signify a major structural transition given the inherent uncertainties in peak fitting. Overall, these findings indicate that the semi-crystalline framework of the PVA matrix remains largely intact, which is in excellent agreement with the stable SAXS profiles discussed earlier.

We noted that the crystallization of 40k chains, starting from a weakly entangled suspension, shows low but non-monotonic sensitivity to LNP content, whereas that of 90k chains from a densely entangled suspension exhibits higher absolute crystallinity that is progressively eroded by nanoparticle addition. This contrast arises fundamentally from the difference in chain mobility. In the weakly entangled 40k system, the fast chain diffusion during film formation allows LNPs to act as efficient, concentration-dependent nucleation sites. The intermediate composition ϕ_c_ = 0.045, where 40k shows a crystallinity maximum, coincides with the well-dispersed LNP state identified in structural analysis. These observations are consistent with reports of supernucleation in polymer–nanoparticle systems [31], where well-dispersed lignin nanoparticles at intermediate concentrations enhance nucleation density, while higher loadings lead to saturation and reduced crystallinity due to aggregation and limited interfacial interactions. Conversely, in the densely entangled 90k system, the slow chains are trapped in a dense network; their restricted mobility prevents them from organizing around LNPs; thus, the nanoparticles act as obstacles that disrupt crystal growth.

### 3.7. Thermal and Optical Properties of PNC Films

**Figure 6:**
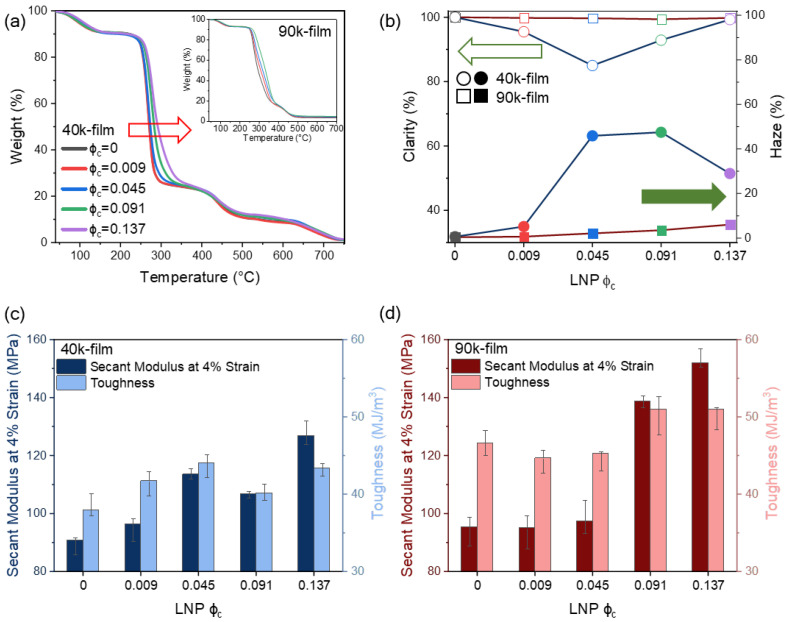
Physical properties of PNC films: thermal stability, optical clarity, and mechanical reinforcement. (**a**) TGA curves for 40k and 90k (inset) systems; the red arrow indicates an increase in decomposition temperature with LNP content. (**b**) Optical properties: clarity (left axis) and haze (right axis) for 40k (circle) and 90k (square) films. The open and filled green arrows indicate the data points corresponding to clarity and haze, respectively. (**c**,**d**) Mechanical properties (Secant modulus at 4% strain and toughness integrated up to 300% strain) for (**c**) 40k and (**d**) 90k films.

To further probe how these structural changes with LNP incorporation are reflected in physical properties, the thermal and optical properties of PNC films were evaluated. Figure 6a shows the TGA results with varying LNP content in 40k PVA. The incorporation of LNPs led to a progressive enhancement in thermal stability, as previously reported [19,20,45]. To quantitatively evaluate this effect, the temperature at 50% weight loss (T_50%_) was utilized as a metric for the major thermal decomposition stage of the PVA matrix. For the 40k system, T_50%_ increased significantly from 270.75 °C for the neat PVA to 291.79 °C at the maximum LNP loading. This substantial increment of approximately 21 °C far exceeds the typical instrumental measurement uncertainty of TGA (approx. ±1–2 °C), confirming a clear and consistent thermal barrier effect of the LNPs. A similar trend was found in 90k PVA (inset of Figure 6a), implying that LNP incorporation does not compromise but increases the thermal stability of the PVA matrix.

Next, the optical properties were examined by measuring haze and clarity of films as a function of LNP content. While the transparency of the films generally decreases with the filler contents, the clarity and haze are known to be sensitive to the microstructure and local heterogeneities on length scales from 100 nm to μm scales. For both molecular weights, neat PVA films remain highly transparent, with haze values near 0% and clarity close to 100%, despite their different absolute crystallinities (X_c_~33% for 40k and 42% for 90k). This indicates that despite the different crystallinity, the characteristic size of the crystalline domains in both films remains well below the Rayleigh-scattering threshold (on the order of λ/10 for visible light); thus, the increased crystallinity mainly reflects a higher number density of nanometric lamellae, rather than the growth of micrometer-scale spherulites that would strongly scatter light [46,47,48]. Increasing molecular weight from 40k to 90k likely thickens individual lamellae, but, due to the higher entanglement density, still constrains the superstructure size to sub-optical dimensions, rendering the 9% difference in X_c_ optically negligible.

For the 40k system, films exhibited a distinct and non-monotonic increase in haze, reaching a peak value of 47.5% (filled blue circle) at intermediate LNP loading (ϕ_c_ = 0.045) from 0.62% (filled black circle) for the neat PVA. This nearly 80-fold increase in haze was accompanied by a pronounced minimum in clarity (85%, open blue circle), directly mirroring the composition where crystallinity shows a modest maximum and LNP dispersion is most uniform. This strong optical response suggests that well-dispersed LNPs in the weakly entangled 40k network not only promote nucleation via rapid chain diffusion but also generate microstructural heterogeneities on length scales closer to the optical scattering window, creating efficient light-scattering domains. This is visually corroborated by the birefringent, mottled texture of crystalline domains observed in polarized optical microscopy (POM) (Figure S8a). In particular, the coexistence of nanometric crystalline domains and LNP-rich regions at intermediate loading is consistent with an intermediate-concentration supernucleation-like regime, in which kinetically facilitated nucleation yields many finely divided domains that perturb the refractive index.

**Figure 7:**
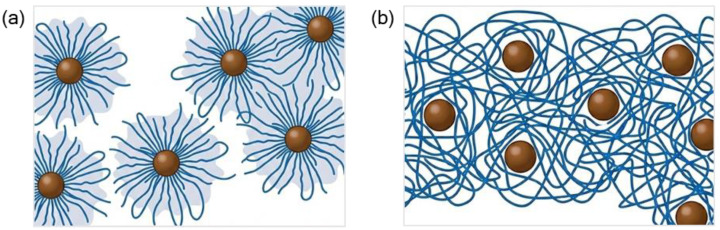
Schematic illustration of PNC network structures at ϕ_c_ = 0.045: (**a**) 40k system (fast kinetics): separated LNP clusters, allowing spherulite formation observed in films. (**b**) 90k system (restricted kinetics): dense chain entanglement fully encapsulates LNPs in the film matrix, promoting homogeneous dispersion.

In contrast, the 90k PVA films showed only minimal changes in haze and clarity with increasing LNP loading, remaining visually uniform across all compositions despite the gradual decrease in crystallinity. The densely entangled 90k network buffers nanoscale structural changes from appearing as macroscopic optical contrast; thus, LNP-induced disruption of crystallinity proceeds mainly through local packing changes and small-scale heterogeneities. Furthermore, the restricted kinetics and network confinement in the 90k films prevent the growth of large scattering centers, allowing the films to preserve high clarity and a homogeneous, featureless morphology as evidenced by POM (Figure S8b). This mechanistic difference is schematically illustrated in Figure 7, which contrasts the nucleation-dominated morphology of the 40k system with the entanglement-stabilized network of the 90k system.

### 3.8. Mechanical Properties

Mechanical properties of the PNC films were evaluated in uniaxial tension using DMA, as shown in Figure 6c,d. Freestanding films with a thickness of approximately 100 μm exhibited high extensibility, sustaining strains up to 300% without failure. Given the non-linear elastic behavior of PVA even at low strains due to molecular and crystalline reorientation, the reported stiffness values represent the secant modulus determined at 4% strain, and toughness was quantified as the energy absorbed up to the 300% strain limit. Since none of the specimens reached failure within this measurement window, this fixed integration interval (up to @ 300%) provides a consistent and objective baseline to compare the energy-dissipation capacity across all LNP compositions. This approach ensures that the observed changes in toughness are a rigorous reflection of the structural reinforcement and improved yield stress, rather than being skewed by variations in ultimate failure strain that occur beyond the instrument’s displacement limit.

In 40k PVA films, the secant modulus increased with LNP content despite the reduced PVA volume fraction, indicating that the stiff, well-bonded LNP phase reinforces the PVA matrix. The tensile strength (represented as the maximum stress at 300% strain) followed a similar upward trend, reaching approximately 16 MPa at the highest loading. It showed a pronounced enhancement at intermediate loading, while toughness displayed a maximum at the same composition before decreasing slightly at higher LNP concentration. This coincidence of modulus and toughness optima with the composition where SAXS, XRD, and optical measurements indicate well-dispersed LNPs and modestly increased crystallinity suggests that nucleation-driven microstructural reinforcement is most effective in the weakly entangled network at intermediate loading. The preservation of high ductility (>300%) alongside enhanced stiffness and strength indicates that LNP incorporation effectively minimizes the typical stiffness–ductility trade-off. This suggests that the LNP–PVA interface facilitates energy dissipation, allowing the composite to achieve higher mechanical reinforcement without triggering premature embrittlement, as evidenced by the sustained plastic flow up to the measurement limit (Figure S9).

In contrast, 90k PVA films exhibited only minor changes in modulus and toughness at low and intermediate LNP concentrations, with properties remaining close to those of neat 90k PVA. At higher LNP contents, the 90k system exhibited a more significant reinforcing effect, with the stress reaching approximately 21 MPa at 300% strain without failure. This abrupt mechanical enhancement suggests the formation of a continuous LNP network, or percolation. While the theoretical percolation threshold (ϕ_c_*) for ideal hard spheres is approximately 0.28–0.30 based on excluded volume theory, the onset of reinforcement at ϕ_c_ = 0.091 in the 90k system suggests an ‘early percolation’ driven by strong inter-particle hydrogen bonding. The densely entangled 90k matrix likely facilitates the structural integration of these LNP clusters, reflecting the development of a rigid, energy-dissipative network that activates mechanical reinforcement once this critical filler threshold is exceeded.

Although the toughness changes are less dramatic than those of the secant modulus, both systems maintain toughness reasonably well as LNP content increases, without a significant brittleness penalty. Overall, the 40k system achieves an effective balance between stiffness and toughness at intermediate LNP loading, making it attractive for applications that require a combination of strength and impact resistance, even though optical properties are compromised. In contrast, the 90k system develops its highest stiffness only at high LNP contents, where the densely entangled matrix supports a more LNP-rich network while preserving optical clarity, which is advantageous for rigidity-dominated transparent applications.

These molecular-weight-dependent trends clarify the role of LNPs and provide a basis for multi-objective optimization of PVA/LNP composites, enabling selection of lignin content and matrix architecture according to specific performance requirements.

## 4. Conclusions

In this study, we demonstrate that lignin can be reproducibly converted into well-defined nanoparticles with tunable sizes and that these uniform LNPs act as effective structural modifiers in PVA matrices. By systematically varying PVA molecular weight, we show how the dispersion state of size-controlled LNPs governs multiscale structure and, in turn, the optical and mechanical properties of LNP-based PNC. Importantly, the introduction of LNPs does not significantly alter the viscosity or flow behavior of the PVA/LNP suspensions, indicating that their primary function is microstructural modulation in the solid state rather than rheological modification in suspension.

In the 40k system, the weakly entangled network accommodates well-dispersed LNPs at intermediate loading, leading to locally enhanced crystallinity and microstructural heterogeneity. This results in a strong, direct coupling between microstructure and optical response: the crystallinity-driven reinforcement simultaneously enhances stiffness and toughness but incurs an optical penalty of increased haze and reduced clarity.

In contrast, the strongly entangled 90k network exhibits a buffered, decoupled behavior. Although increasing LNP content progressively disrupts crystalline order, this LNP-induced disruption does not strongly translate into additional scattering centers. Consequently, the 90k system largely preserves optical transparency even at high loadings, with mechanical reinforcement emerging only when a rigid, energy-dissipative nanofiller network develops. This highlights the decisive role of entanglement in determining how LNP-induced structural changes are expressed in the optical performance: in 90k networks, the densely entangled matrix effectively buffers and filters nanoscale perturbations from propagating to macroscopic length scales.

Overall, this molecular-weight-dependent design framework provides information for rational optimization of LNP-based PNC by controlling LNP size, concentration, and PVA entanglement density to achieve targeted combinations of mechanical robustness and optical performance. More broadly, the demonstration that lignin can be transformed into well-defined, size-controlled nanoparticles, and that their dispersion and resulting microstructural modulation can be systematically governed by matrix entanglement, provides a fundamental basis for exploiting lignin as a sustainable nanofiller. This entanglement-mediated design framework offers a strategic pathway to influence the length scales of crystalline heterogeneities and interfacial energy dissipation. Such a framework can be universally extended to other water-soluble or hydrogen-bonding polymer–LNP systems (e.g., cellulose derivatives, poly(ethylene oxide)), advancing the development of multifunctional bio-based nanocomposites with tailored mechanical and optical properties.

## Data Availability

The original contributions presented in the study are included in the article/Supplementary Materials. Further inquiries can be directed to the corresponding authors.

## Supplementary Materials

The following supporting information can be downloaded at: https://www.mdpi.com/article/10.3390/polym18060691/s1, Note S1: Theoretical estimation of the LNP hydrodynamic contribution to suspension viscosity. Figure S1: Visual appearance of LNP dispersions in water without PVA at increasing concentrations. Figure S2: Long-term stability of LNP dispersions in PVA at different molecular weights. Figure S3: Raw data of rheological and SAXS for PVA/LNP suspensions. Figure S4: Raw rheological and SAXS data for PVA/LNP suspensions in a constant-solid content system. Figure S5: FT-IR spectra of the PNC films. Figure S6: XRD patterns with peak deconvolution for 40k PNC films. Figure S7: XRD patterns with peak deconvolution for 90k PNC films. Figure S8: Polarized optical microscopy (POM) images of PNC films at ϕ_c_ = 0.045. Figure S9. Representative stress–strain curves of PVA/LNP composite films at ϕ_c_ = 0.137.
