# Supplementary material for: Entanglement-Mediated Dispersion of Lignin Nanoparticles in PVA Networks for Transparent and Tough Bio-Composites
Source: Polymers (Basel). 2026 Mar 12;18(6):691. doi: 10.3390/polym18060691 (PMC13029828; doi:10.3390/polym18060691)
Supplement: Supplementary file 1 [file polymers-18-00691-s001.zip › polymers-4171296-supplementary.pdf]

Supplementary Materials

Entanglement-Mediated Dispersion of Lignin Nanoparticles in PVA Networks for Transparent and Tough Bio-Composites

In Jun Lee[1, 2], *and So Youn Kim*[1, *]

[1]Department of Chemical and Biological Engineering, Institute of Chemical Processes, Seoul National University, Seoul 08826, Republic of Korea

[2]Hanwha Solutions Chemical Division Corporation, Research and Development Institute, Daejeon, 34128, Republic of Korea.

Corresponding Author. Tel: +82 02 880 1505. E-mail: soyounkim@snu.ac.kr (S. Y. K.)

1. Supplementary Notes

Note S1. Theoretical estimation of the LNP hydrodynamic contribution to suspension viscosity

To verift that the purely hydrodynamic contribution of the LNPs to the macroscopic suspension viscosity is negligible, the Huggins equation was applied:

$$\frac{\eta}{\eta_0} = 1 + [\eta]\phi_c + k_H[\eta]^2{\phi_c}^2$$

Where η is the viscosity of the suspension, η_0 is the viscosity of the polymer matrix, [η] is the intrinsic viscosity shape factor, ϕ_c is the particle volume fraction, and k_H is the Huggins constant.

1. Shape Factor ([η]): Since the synthesized LNPs via self-precipitation are highly isotropic and spherical (aspect ratio ~ 1), the theoretical Einstein shape factor for hard spheres ([η]= 2.5) was utilized.
2. Volume Fraction (ϕ): At the maximum LNP loading (15 wt% relative to the 5 wt% total solids), the mass fraction of LNPs in the entire aqueous suspension is 0.75 wt%. A density of Kraft lignin is 1.35 g/cm^3 and water is about 1.00 g/cm^3, the maximum volume fraction is calculated to be ϕ ~ 0.00568.
3. Calculation: A typical Huggins constant for well-dispersed systems (k_H ~ 0.4) was assumed. It is worth noting that because the volume fraction is extremely small (ϕ ~ 0.00568), the second-order term ($k_H[\eta]^2\phi^2 \sim 10^{-5}$) is mathematically negligible regardless of the specific k_H value. The relative viscosity is predominantly dictated by the first-order Einstein term:

$$\frac{\eta}{\eta_0} = 1 + 2.5(0.00568) + 0.4(2.5)^2(0.00568)^2 \sim 1.014$$

This calculation demonstrates that the hydrodynamic presence of the LNPs increases the total macroscopic viscosity by merely ~1.4%, strictly remaining below the 5% margin of error and confirming that the rheological signature is predominantly governed by the polymer network.

2. Supplementary Figures

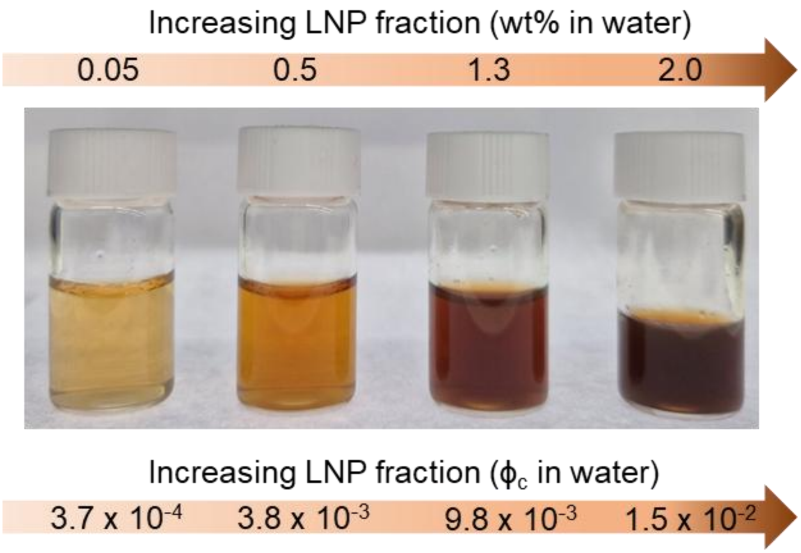

Figure S1. Visual appearance of LNP dispersions in water without PVA at increasing concentrations. All samples are clear and stable without aggregation, demonstrating excellent colloidal stability.

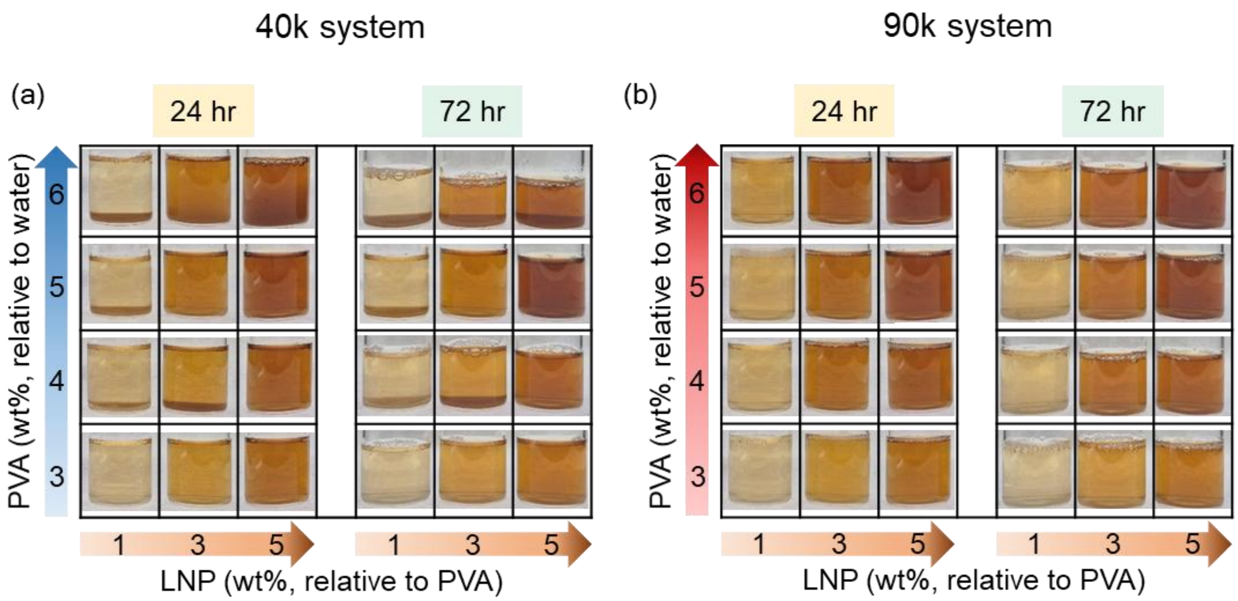

Figure S2. Long-term stability of LNP dispersions in PVA at different molecular weights. (a) 40k PVA system and (b) 90k PVA system.

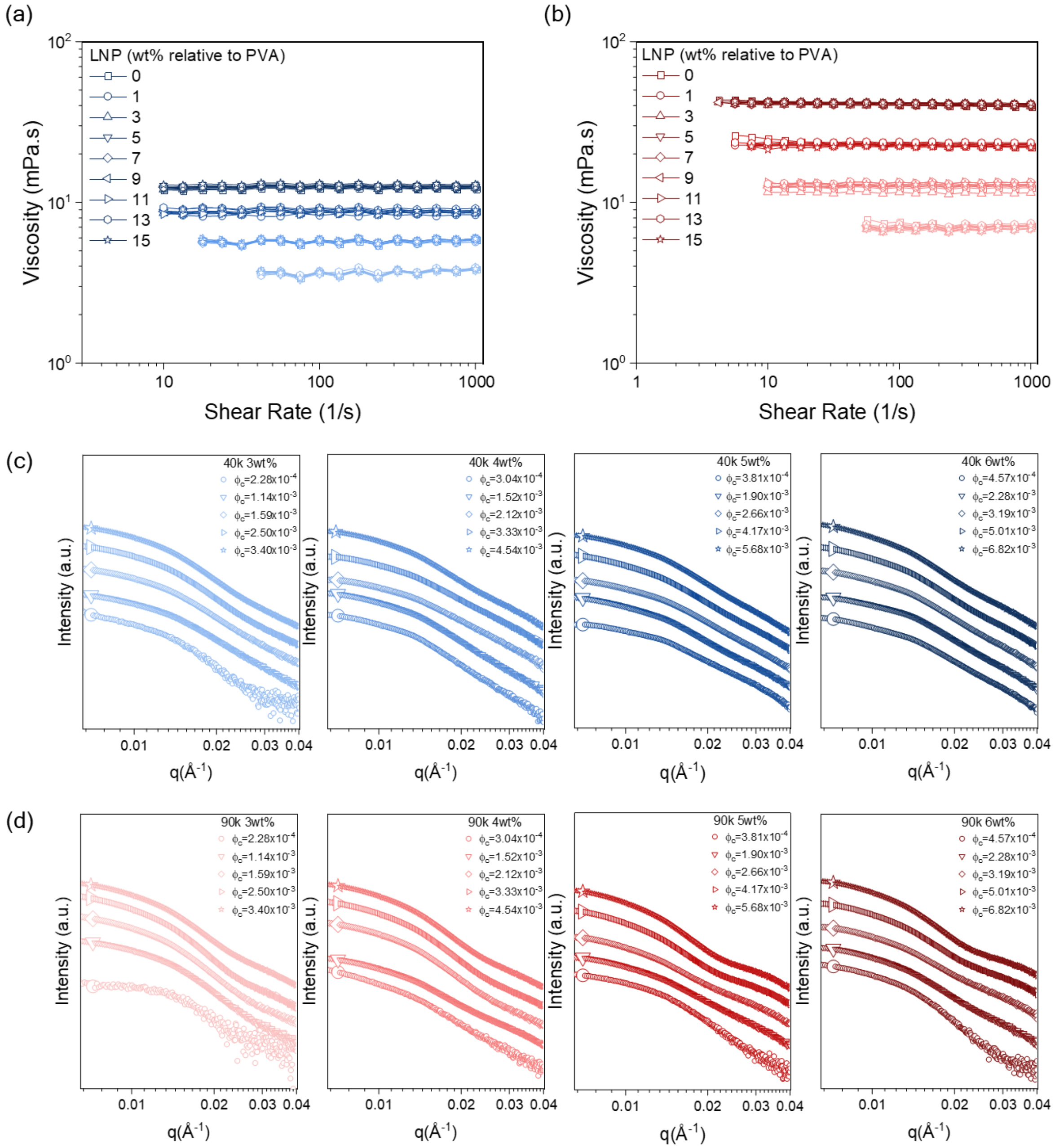

Figure S3. Raw data of rheological and SAXS for PVA/LNP suspensions. (a) Shear viscosity for 40k system at varying LNP wt% (relative to PVA, 3 to 6 wt%). (b) Shear viscosity for 90k system at varying LNP wt% (relative to PVA, 3 to 6 wt%). (c) SAXS intensity profiles for 40k systems. (d) SAXS intensity profiles for 90k systems

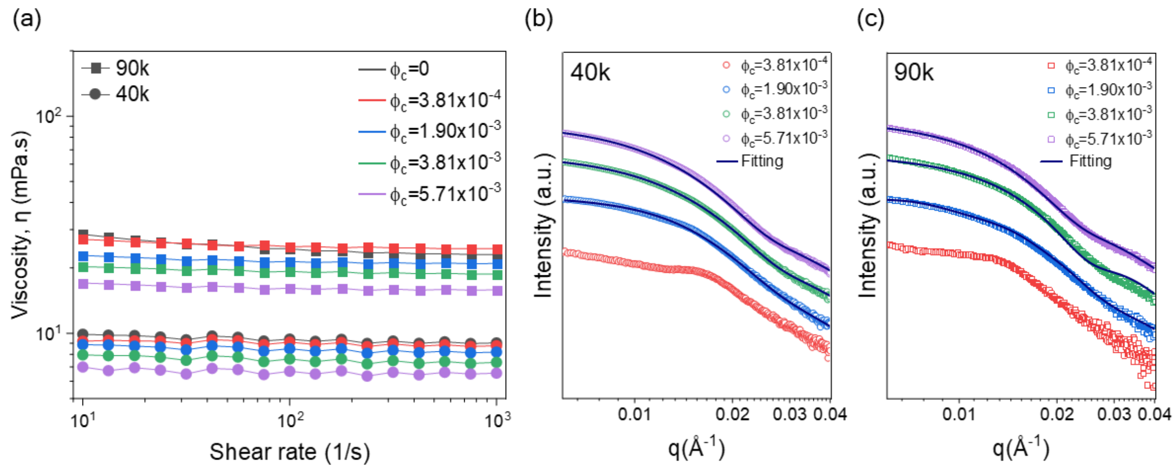

Figure S4. Raw rheological and SAXS data for PVA/LNP suspensions in constant solid contents system. (a) Shear viscosity for 40k (circle) and 90k (square) system. (b) Form factor fitting for 40k system; blue (diameter = 32.4nm, std. dev. = 0.26), green (26.8nm, 0.35), purple (27.2nm, 0.37), (c) Form factor fitting for 90k system: blue (28.0nm, 0.32), green (32.0nm, 0.21), purple (39.6nm, 0.23)

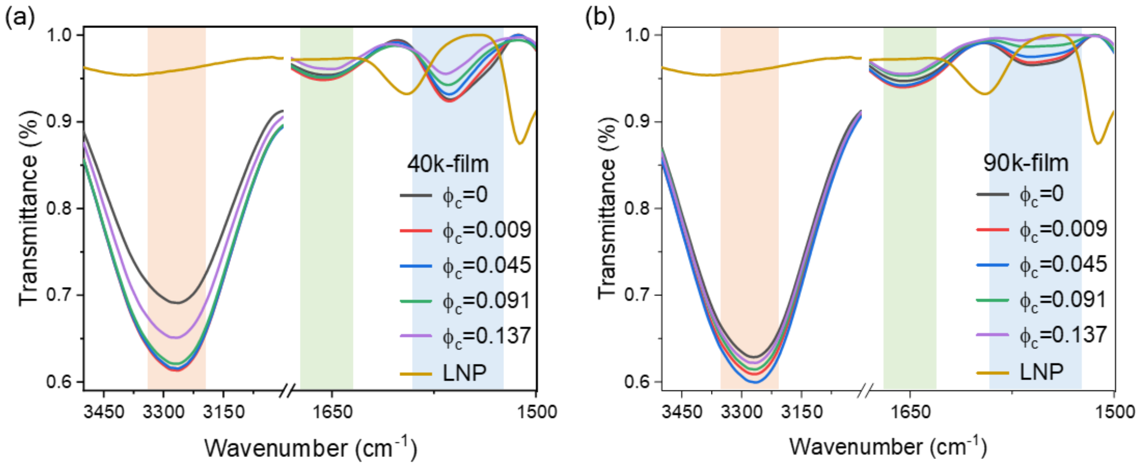

Figure S5. FT-IR spectra of the PNC films. (a) 40k system film. (b) 90k system film

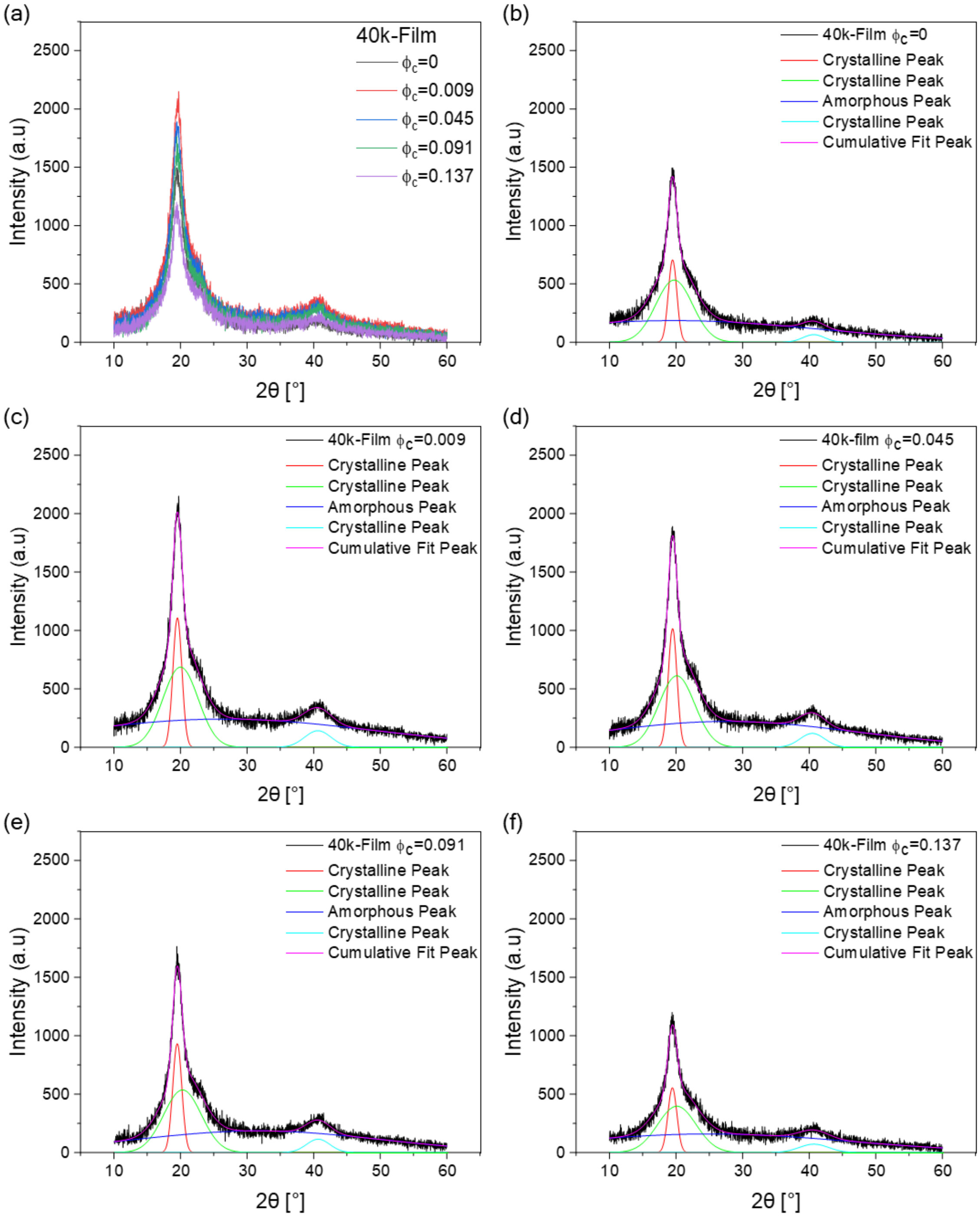

Figure S6. XRD patterns with peak deconvolution for 40k PNC films.
(a-e) Deconvoluted spectra at varying LNP content.

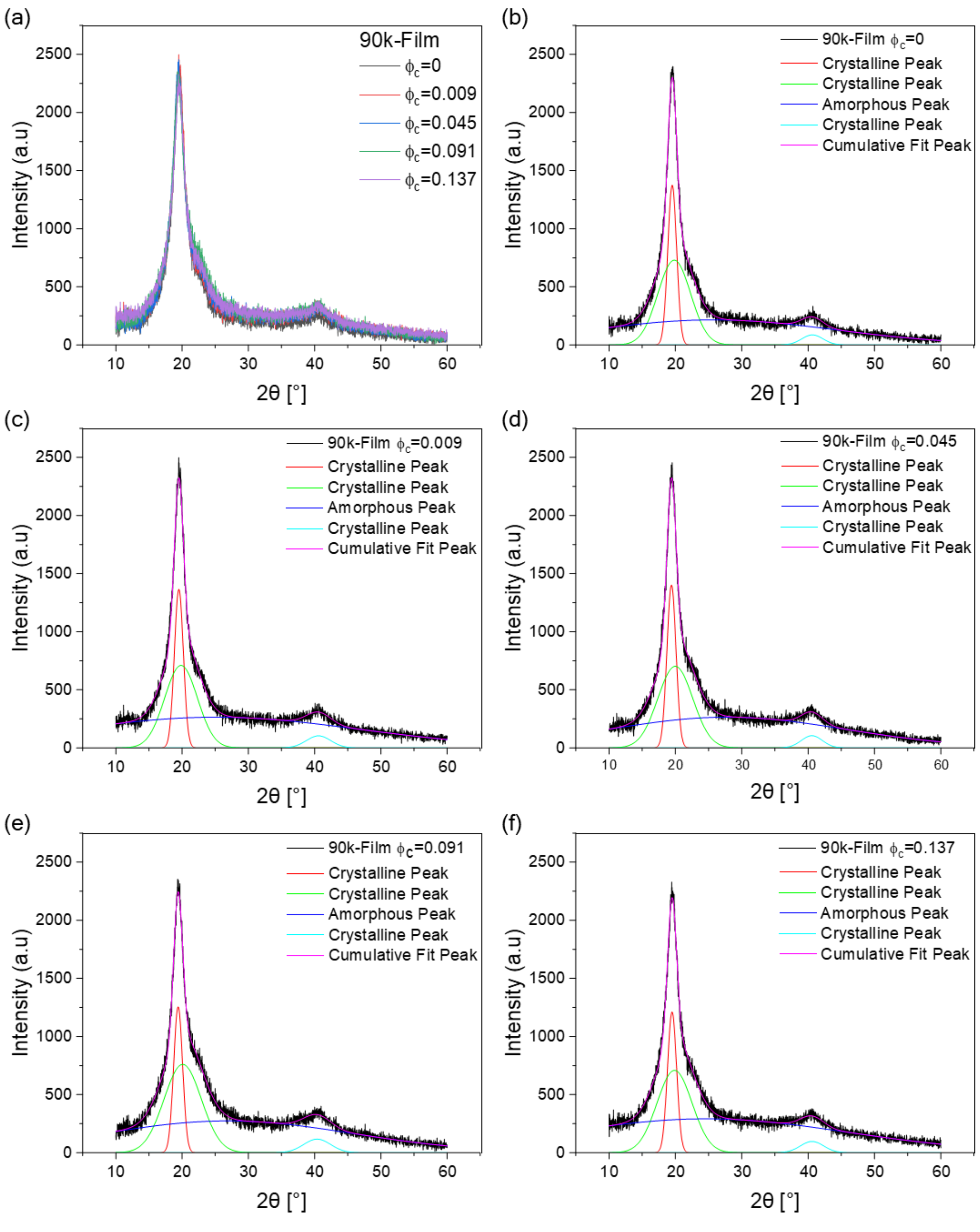

Figure S7. XRD patterns with peak deconvolution for 90k PNC films.
(a-e) Deconvoluted spectra at varying LNP content.

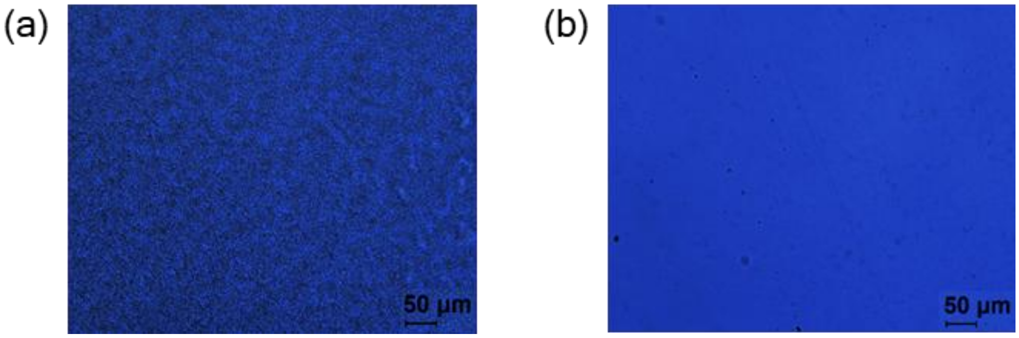

Figure S8. Polarized optical microscopy (POM) images of PNC films at ϕ_c = 0.045. (a) 40k PNC : Birefringent, mottled texture of crystalline domains. (b) 90k PNC : Homogeneous and featureless morphology

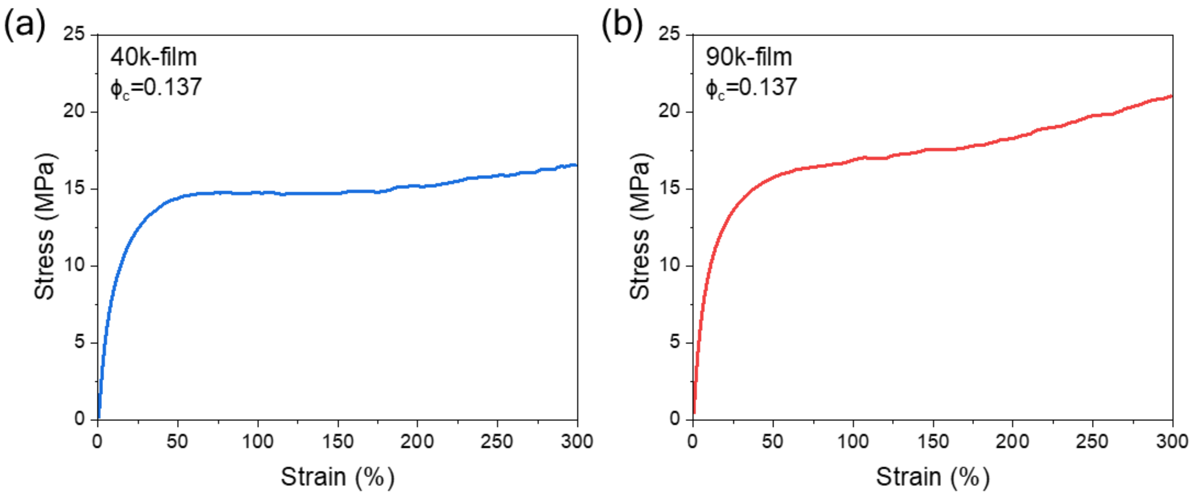

Figure S9. Representative Stress-Strain Curves of PVA/LNP Composites films at ϕ_c = 0.137. Both (a) 40k and (b) 90k systems exhibited high extensibility, sustaining strains up to 300% without failure. Due to the displacement limit of the DMA instrument, measurements were terminated at 300% strain; however, the lack of fracture confirms that LNP incorporation does not induce premature embrittlement. The maximum stress reached approximately 16 MPa for the 40k system and 21 MPa for the 90k system at the 300% strain threshold.
